# Exploring the Needs of Health Professionals for a Type 2 Diabetes Remote Patient Monitoring Dashboard for Personalized Care: Focus Group Study

**DOI:** 10.2196/84894

**Published:** 2026-03-30

**Authors:** Chiara Lansink, Nienke Beerlage-de Jong, Eclaire Hietbrink, Anouk Middelweerd, Gozewijn Dirk Laverman

**Affiliations:** 1 Department of Biomedical Signals and Systems, Univerisity of Twente, Enschede, 7500 AE, The Netherlands, 31 53 489 5753; 2 Department of Internal Medicine/Nephrology, Ziekenhuisgroep Twente, Almelo, The Netherlands; 3 Section of Psychology, Health and Technology, University of Twente, Enschede, The Netherlands

**Keywords:** type 2 diabetes mellitus, clinical decision support system, health information technology, health personnel, patient-centered care, digital health, blended care, dashboard

## Abstract

**Background:**

Effective management of type 2 diabetes mellitus (T2DM) requires monitoring clinical parameters like blood glucose and medication, alongside lifestyle factors such as diet and physical activity. Decision support tools, including dashboards and shared decision-making tools, help with medication adjustments, glucose monitoring, and lifestyle. However, systems rarely integrate home-monitored lifestyle data with personalized guidance and rarely facilitate collaborative goal setting for behavior change. As a result, health care professionals (HCPs) are limited in their ability to support patients’ medical and lifestyle management. Blended care, combining in-person consultations with digital monitoring of patient data, can help bridge this gap by providing structured information and data-driven insights to support diabetes management.

**Objective:**

The study aims to identify HCPs’ requirements for a remote monitoring dashboard for people with diabetes that integrates clinical and home-monitored lifestyle data, supporting personalized, patient-centered care and collaborative goal setting in blended T2DM management.

**Methods:**

A qualitative study was conducted using 2 interactive focus group sessions with HCPs involved in the treatment of T2DM. Focus group participants shared experiences, identified practical needs, and collaboratively defined requirements for a dashboard to support personalized diabetes management. Transcripts were coded to identify recurring themes and ideas, which were then consolidated into distinct requirements. Requirements were labeled with an identification code (ID) and categorized in accordance with the FICS framework, distinguishing 4 types of design requirements: functions and events (F), interaction and usability (I), content and structure (C), and style and aesthetics (S). Prioritization of requirements was performed using the must/should/could/will not have method.

**Results:**

In total, 9 HCPs participated in 2 focus groups, each lasting approximately 1.5 hours. A total of 50 requirements for a T2DM dashboard were identified. Of these, 31 (62.0%) were functions and events (F), 9 (18.0%) related to interaction and usability (I), 7 (14.0%) concerned content and structure (C), and 3 (6.0%) pertained to style and aesthetics (S). The participants expressed the need for a dashboard that incorporates data-driven lifestyle (eg, physical activity and nutrition) with visual trend analysis and integration of psychosocial aspects. They also emphasized the importance of visualizing how nutrition, physical activity, and medication interact to influence glucose values. In addition, participants expressed the wish for a home screen that provides a quick overview, with the option to click through to more detailed views (eg, per day, week, or month).

**Conclusions:**

The findings demonstrate a demand among HCPs for an integrated dashboard that combines clinical, lifestyle data, and psychological data to support personalized, patient-centered care. By linking lifestyle behaviors with glucose outcomes, such a tool could support collaborative goal setting, strengthen blended care pathways, and promote data-driven diabetes management. The findings provide guidance for the design of digital health interventions tailored to the needs of HCPs.

## Introduction

Diabetes is one of the most common chronic diseases worldwide [1]. In 2022, approximately 1.2 million people in the Netherlands had diabetes, with the majority being diagnosed with type 2 diabetes mellitus (T2DM) [2,3], and this number is expected to rise to 1.5 million by 2040 [2]. This places a growing burden on health care systems and increases the need for effective prevention and management strategies [4]. T2DM is strongly influenced by lifestyle factors, with unhealthy behaviors playing a major role in its development and progress [5]. Maintaining a healthy lifestyle, including regular physical activity and balanced nutrition [6-8], as well as stress management and adequate sleep [9,10], is crucial for glycemic control and overall health outcomes. However, despite the well-known benefits of a healthy lifestyle, recent studies showed that 49% of the Dutch population did not meet the recommended physical activity guidelines [11,12], and similar patterns are observed in persons with diabetes. Adherence to both nutritional and physical activity recommendations is low, with only a small proportion of persons with diabetes meeting the guidelines [13-16].

Addressing lifestyle as part of treatment is emphasized in the diabetes guidelines [17,18]. Health care professionals (HCPs) are expected to support persons with diabetes in adopting healthier behaviors by assessing lifestyle factors, offering guidance, and helping individuals prioritize achievable changes [19]. In this context, HCPs serve as the critical link in translating lifestyle recommendations into practical advice that persons with diabetes can apply in daily life. However, routine practice consultations often focus primarily on glucose levels and evaluation of medication effects on parameters such as blood pressure and cholesterol [20,21], with limited attention to lifestyle counseling due to time constraints [20,22].

To help address these challenges, digital tools have been increasingly explored as supportive tools in diabetes care. Reviews highlight the potential of mobile health apps, continuous glucose sensors, and activity trackers to support lifestyle changes and diabetes self-management [23-27]. In addition to monitoring lifestyle, decision-support systems and remote persons with diabetes monitoring have been shown to help reduce pressure on health care services by decreasing outpatient visits and consultation times and reducing hospital admissions [28,29]. At the same time, HCPs consistently highlight the value of face-to-face contact [30-32], suggesting that digital solutions are most useful when embedded within blended care models. Such models, in which digital tools are integrated into in-person consultations, are supported by evidence, indicating that digital innovations are more effective when complemented by a human component [33-37].

One particularly promising digital tool to be used in such blended care is a dashboard that facilitates shared decision-making by presenting relevant health data in a structured and visually accessible format. Shared decision-making allows HCPs and persons with diabetes to jointly tailor care plans to individual preferences and values [38]. To the best of our knowledge, no existing dashboard integrates lifestyle behaviors such as diet and physical activity with clinical metrics like continuous glucose monitoring into personalized lifestyle advice in a data-driven way. This hinders the delivery of data-driven care that goes beyond glucose regulation alone. The development of a dashboard that combines these data streams into tailored recommendations could therefore provide a practical blended care solution. It could strengthen shared decision-making and support personalized diabetes management in routine care.

Therefore, the objective of this study was to identify and determine the requirements of HCPs for a remote patient monitoring dashboard that visualizes health data (eg, glucose regulation, physical activity, and nutrition) to support personalized blended care and shared decision-making. Through focus groups, we investigated which information HCPs consider most relevant, how they prefer it to be presented, and what additional data would make the dashboard most clinically useful. The ultimate goal is to enable HCPs and persons with diabetes to collaboratively set personalized lifestyle goals and make informed treatment decisions, thereby facilitating more individualized care within blended care settings.

## Methods

### Design

This study used a stepwise approach, collecting data through 2 interactive focus groups. These sessions facilitated both the sharing of experiences and collaborative identification of practical needs for the dashboard, making this approach well-suited for designing user-centered digital health interventions. The focus groups were conducted following Raats’ guidelines for health care settings [39].

### Ethical Considerations

The study was reviewed and approved by the ethics committee of the Faculty of Behavioural, Management and Social Sciences at the University of Twente (reference: 240227). Written informed consent was obtained from all participants prior to the initiation of the focus groups, and they were fully informed about the purpose of the study. Participants’ privacy and confidentiality were strictly maintained throughout the research. No compensation was provided for participants. At the beginning of each session, participants were reminded that it would be recorded.

### Participants and Recruitment

We used purposive sampling to recruit HCPs involved in T2DM care across secondary and primary care in the Twente region (Netherlands). In total, 27 HCPs were invited across both sessions. In the first focus group, 8 HCPs from secondary care, together with a lifestyle coach, were invited to participate in an initial focus group, aimed at exploring needs and challenges in secondary care. All of them were welcome to participate in both sessions. In total, 16 HCPs were approached for the second focus group, including professionals from the 2 regional hospitals and primary care providers. This study had an exploratory qualitative design. The aim was to generate new insights rather than to reach statistical generalizability. Consequently, a smaller yet information-rich sample of diabetes nurses was considered methodologically appropriate [40].

### Data Collection

**Figure 1.**
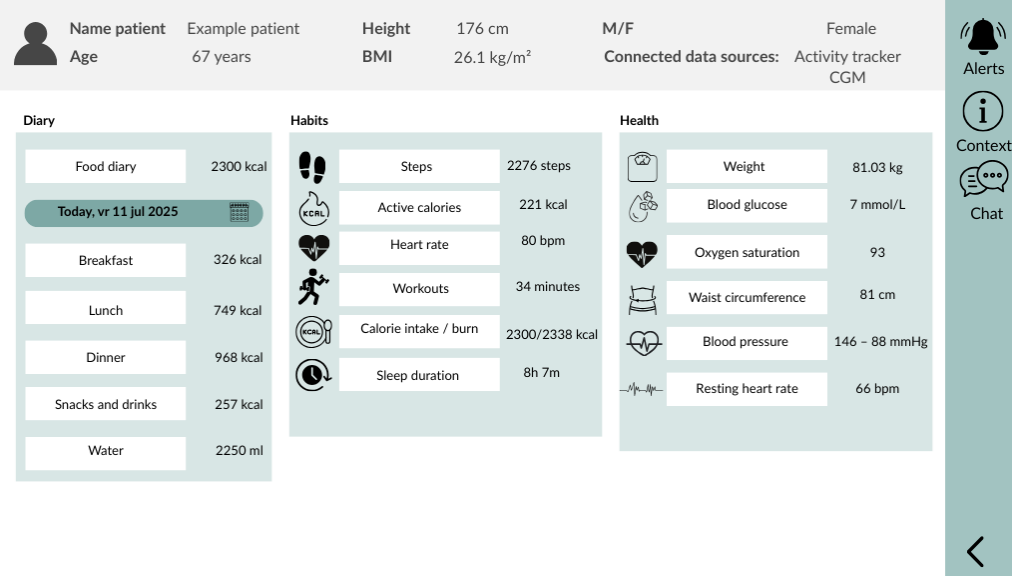
Visualization of a mock-up dashboard that was shown to the health care professionals during the second focus group.

Two 1.5-hour focus groups were conducted following established guidelines for health care–based group discussions and using a structured requirements development approach. Each session began with introductions and followed a predefined protocol covering current monitoring practices, desired data visualization, and workflow integration. In the first session, participants mapped the current and ideal care pathways using a hypothetical referral case and identified corresponding dashboard needs. They then individually listed and ranked their requirements, which were jointly reviewed and prioritized using the must/should/could/will not have (MoSCoW) approach [41]. Based on requirements obtained during the first session, a mock-up dashboard was created (Figure 1).

In the second session, participants designed an initial dashboard by selecting and ranking essential parameters, compared these with previously identified requirements, and provided feedback on an interactive mock-up. The session also explored challenges and opportunities for transmural (primary-secondary) collaboration and the potential role of the dashboard in blended care. The researchers closely monitored group dynamics and ensured a safe environment in which participants could speak up and discuss differences in opinion.

### Data Analysis

Audio recordings were transcribed verbatim using Amberscript and corrected manually. Session summaries were member-checked by participants. We applied a 9-step requirements development method [42]: familiarization, selection of relevant quotes, attribute assignment, grouping and refinement, translation into preliminary requirements, independent review, and consensus building. Relevant quotes were identified using Braun’s selection criteria [43]. An independent researcher (NB-J) provided a second opinion review to strengthen the reliability of the analysis.

To organize the final set of requirements, we used the FICS structure to present results from a design-oriented perspective [44]. This framework distinguishes 4 types of design requirements: functions and events (F), referring to the core features needed for the dashboard to operate; interaction and usability (I), relating to user experience and accessibility; content and structure (C), focusing on how information is organized and presented; and style and aesthetics (S), addressing visual design and appeal. Prioritization of the resulting requirements followed the MoSCoW categories; in the first focus group, these were assigned directly by participants, and in the second focus group, they were interpreted and categorized by the researcher (CL).

## Results

### Characteristics of the Participants

In total, 11 participants agreed to take part in the focus groups. Due to illness (n=2) and personal circumstances (n=1), eventually, 9 participants attended the sessions, one of whom joined both sessions (n=10). Each session included 5 participants. The group consisted of 6 female and 3 male participants, including 3 diabetes nurses, 1 diabetes specialist nurse, 3 internists, and 2 general practitioner practice assistants. Of these, 2 participants were from primary care and 7 from secondary care. The 3 participating internists each had a different subspecialty: 1 in endocrinology, 1 in nephrology, and 1 in vascular medicine. An overview of the participants’ characteristics is provided in Table 1.

**Table 1.** Unique focus group participants (N=9)^a^.

Characteristics		Participants, n (%)
Sex		
	Female	6 (66.7)
	Male	3 (33.3)
Profession		
	Diabetes nurse	3 (33.3)
	Diabetes nurse practitioner	1 (11.1)
	Internist	3 (33.3)
	General practitioner practice assistant	2 (22.2)
Type of care		
	Primary care	2 (22.2)
	Secondary care	7 (77.7)
Years of experience in diabetes care		
	0‐10	5 (55.6)
	11‐20	1 (11.1)
	20+	3 (33.3)

a In total, 1 participant joined both focus groups, resulting in a total of 9 unique participants.

### Overview of the Total Requirements

A total of 50 requirements were identified. These requirements were categorized into 4 main groups (see also Table 2): 31 (62.0%) functions and events (F) requirements, 9 (18.0%) interaction and usability (I) requirements, 7 (14.0%) content and structure (C) requirements, and 3 (6.0%) style and aesthetics (S) requirements.

**Table 2.** The number of formulated requirements as a result of the focus groups per FICS^a^ category for overarching, dietary intake, physical activity, glucose values, social domain, and additional parameters.

	Overall system, n (%)	Dietary intake, n (%)	Physical activity, n (%)	Glucose values, n (%)	Psychosocial aspects, n (%)	Additional parameters, n (%)	Total, n (%)
Functions and events	10 (20.0)	3 (6.0)	3 (6.0)	10 (20.0)	3 (6.0)	2 (4.0)	31 (62.0)
Interaction and usability	5 (10.0)	0 (0.0)	1 (2.0)	3 (6.0)	0 (0.0)	0 (0.0)	9 (18.0)
Content and structure	1 (2.0)	2 (4.0)	0 (0.0)	0 (0.0)	1 (2.0)	3 (6.0)	7 (14.0)
Style and aesthetics	2 (4.0)	0 (0.0)	0 (0.0)	1 (2.0)	0 (0.0)	0 (0.0)	3 (6.0)
Total	18 (36.0)	5 (10.0)	4 (8.0)	14 (28.0)	4 (8.0)	5 (10.0)	50 (100.0)

a FICS: Functions and events, interaction and usability, content and structure, and style aesthetics.

The requirements covered overall system requirements, including accessibility of the dashboard as well as requirements specifying the design of the dashboard. They also included specific requirements for the monitoring of dietary intake, physical activity, glucose values, psychosocial aspects of living with T2DM, and additional parameters (eg, laboratory results and sleep).

A total of 37 (74.0%) requirements were classified as “must have” or “should have,” indicating their essential role in the dashboard’s core functionality. The remaining 13 (26.0%) requirements fell into the “could have” or “will not have” categories, meaning that they were not essential for basic functionality or were excluded from this phase to avoid overcomplicating the design. An overview of all requirements and their classification can be found in Tables 3-8.

**Table 3.** Requirements regarding the overall system^a^.

ID	Requirement	Justification	Must have	Should have	Could have	Will not have
Functions and events						
F1	The dashboard is accessible to all HCPs^b^ involved in diabetes management (eg, diabetes nurses, primary care physicians, and specialists).	Diabetes management often involves multiple HCPs.	✓			
F2	The dashboard supports integration with multiple health apps.	This is considered important for long-term sustainability and broader adoption of the dashboard.				✓
F3	There is a home screen.	A home screen provides fast information to the HCPs.	✓			
F4	The home screen includes data about glucose values, nutritional intake, physical activity, and medication use.	This information provides a clear century point for the consultations.	✓			
F5	The home screen includes a clickable structure that allows HCPs to easily navigate to more detailed data.	A clickable structure keeps the balance between overview and depth.		✓		
F6	Persons with diabetes can be monitored over a specific period.	Allows HCPs to review specific time frames to evaluate treatment effectiveness.	✓			
F7	Integrated chat feature allows persons with diabetes to easily contact health care providers and vice versa.	Improves communication and enables timely responses between consultations.			✓	
F8	The chat feature automatically sends notifications to HCPs.	Ensures messages are seen promptly.			✓	
F9	Automated messages are sent to persons with diabetes based on their physical activity, nutrition, and glucose levels using artificial intelligence.	This represents potential future improvement for automated personalized diabetes care.				✓
F10	Data can be anonymized for use in training.	Enables the use of real cases for education and system testing while protecting privacy and complying with ethical standards.			✓	
Interaction and usability						
I1	The dashboard can integrate into existing software systems.	This supports smooth adoption in daily practice.	✓			
I2	The dashboard can connect with multiple electronic health record systems.	This ensures interoperability across primary care and secondary care HCPs.	✓			
I3	The dashboard provides easily understandable information at a glance.	Quick and clear insights support engagement without being overwhelmed by data.	✓			
I4	The dashboard enables HCPs to select which parameters are visible, allowing for customization based on agreements with the person with diabetes.	Tailored dashboards prevent information overload.		✓		
I5	Navigation within the dashboard follows a logical and consistent structure.	Navigating in the dashboard should align with clinical workflows.	✓			
Content and structure						
C1	The health record of a person with diabetes is accessible for different HCPs involved, meaning that the health record is accessible across care domains and pathways.	Promotes interdisciplinary collaboration.		✓		
Style and aesthetics						
S1	The home screen can be personalized by HCP, based on their role, specialization, or preferences.	Not all HCPs need the same information; for example, an internist has different priorities than a nurse. Personalization increases the relevance of the information displayed.		✓		
S2	The dashboard includes an overview of historic data of persons with diabetes in an overview per day, per week, and per month.	To see the progression over time.		✓		

a Requirements are categorized as functions and events (F), interaction and usability (I), content and structure (C), and style and aesthetics (S). Each has an identification code (ID) in the first column, matching references in the main text.

b HCP: health care professional.

**Table 4.** Requirements regarding dietary intake^a^.

ID	Requirement	Justification	Must have	Should have	Could have	Will not have
Functions and events						
F11	The dashboard enables the display of an overview of the consumed carbohydrates, amount of fat, and proteins.	This overview allows HCPs^b^ to quickly assess general nutritional intake.	✓			
F12	Data output (see F11) is stored per day and presented for breakfast, lunch, dinner, in-between meals, and total carbohydrate intake.	To gain insight into carbohydrate distribution over the day.		✓		
F13	The dashboard provides HCPs with basic nutritional information and practical guidelines, such as the percentage of dietary reference intake for relevant nutrients (see F11).	To enable HCPs to confidently address general nutrition questions.	✓			
Content and structure						
C2	Data output is presented in grams per day.	For each product, the amount of, for example, carbohydrates in grams per day should be calculated, based on nutritional values per 100 grams of the food item as noted in the Dutch Food Composition Table.	✓			
C3	Nutritional data are displayed in detail, breaking it down into carbohydrates, proteins, fat, fluid intake, and alcohol consumption.	Detailed breakdowns provide deeper insights into specific nutritional behaviors.	✓			

a Requirements are categorized as functions and events (F) and content and structure (C). Each has an identification code (ID) in the first column, matching references in the main text.

b HCP: health care professional.

**Table 5.** Requirements regarding physical activity^a^.

ID	Requirement	Justification	Must have	Should have	Could have	Will not have
Functions and events						
F14	The dashboard can display MVPA^b^ levels and step count.	MVPA provides insight into activity intensity, which is essential for evaluating whether persons with diabetes meet physical activity guidelines.	✓			
F15	The dashboard displays physical activity through step counts and MVPA for daily, weekly, monthly, and yearly periods.	Insight into long-term trends of physical activity data and variations in activity levels over time allows HCP^c^ for more personalized monitoring.	✓			
F16	The dashboard displays all types of physical activity, including sports do not capture through step count.	Provides a more complete picture of the activity level.		✓		
Interaction and usability						
I6	Persons with diabetes can manually input physical activities that are not automatically tracked by an activity device.	Ensures untracked but relevant activities are included.		✓		

a Requirements are categorized as functions and events (F) and interaction and usability (I). Each has an identification code (ID) in the first column, matching references in the main text.

b MVPA: moderate to vigorous physical activity.

c HCP: health care professional.

**Table 6.** Requirements for glucose values^a^.

ID	Requirement	Justification	Must have	Should have	Could have	Will not have
Functions and events						
F17	The dashboards display the percentage of time in range, time below range, and time above range.	This allows HCPs^b^ to quickly assess glucose levels.	✓			
F18	The dashboard displays the glucose values throughout the day and the progress over time.	This insight helps HCPs to easily track glucose control of people with T2DM^c^.	✓			
F19	The dashboard displays glucose variability during the day.	This information helps HCPs identify specific times or behaviors that may contribute to instability.	✓			
F20	The dashboard can support artificial intelligence–based adjustments of medication based on glucose sensor data.	This represents a potential future improvement for automated personalized diabetes care.				✓
F21	The dashboard can synchronize with various glucose monitoring devices.	This makes it usable for a broader target population.			✓	
F22	Blood glucose value thresholds are adjustable.	The thresholds can differ per person with diabetes.	✓			
F23	The dashboard records and displays how often a person with diabetes scans their glucose sensor.	This information helps HCPs identify potential issues such as excessive scanning.			✓	
F24	Notifications with general advice are sent automatically from the dashboard to the person with diabetes when glucose values are out of range.	Notifications with advice help persons with diabetes quickly manage abnormal glucose levels.			✓	
F25	Alerts are only sent to HCPs in specific, well-defined cases.	HCPs should not be overwhelmed with notifications.	✓			
F26	Alerts forwarded to HCPs are filtered according to predefined clinical protocols.	Unfiltered alert risk will overwhelm HCPs with unnecessary notifications, which may reduce efficiency and contribute to alarm fatigue.		✓		
Interaction and usability						
I7	The dashboard provides a clickable option on the home screen to access a detailed view of glucose data.	Offering easy access to a more detailed view of glucose data (F17 and F18).		✓		
I8	The dashboard supports the wireless transfer of glucose data from the devices of persons with diabetes.	Wireless transfer ensures that the HCPs have access to glucose data.	✓			
I9	Persons with diabetes are considered primarily responsible for acting based on their glucose data.	HCPs cannot continuously present to monitor or respond to real-time fluctuations.	✓			
Style and aesthetics						
S3	The detailed glucose overview clearly displays how activity, nutrition, and medication influence blood glucose levels in an easy-to-understand way.	Presenting glucose values in relation to this provides deeper insight into glucose regulation.		✓		

a Requirements are categorized as functions and events (F), interaction and usability (I), and style and aesthetics (S). Each has an identification code (ID) in the first column, matching references in the main text.

b HCP: health care professional.

c T2DM: type 2 diabetes mellitus.

**Table 7.** Requirements for psychosocial aspects^a^.

ID	Requirement	Justification	Must have	Should have	Could have	Will not have
Functions and events						
F27	The dashboard includes information on relevant social support within the region.	HCPs^b^ lack visibility in available social resources. Integration into the dashboard makes this insightful and increases the likelihood of being referred to it.			✓	
F28	The dashboard includes a visualization of the stress levels of persons with diabetes.	Visually displaying stress can help HCPs get an overview of the impact of psychological factors on glucose regulation.		✓		
F29	Persons with diabetes can add notes to their measurements for health care providers.	Notes provide context for the measurements.			✓	
Content and structure						
C4	The dashboard displays the positive health spider web.	This ensures that HCPs can easily interpret trends in well-being.		✓		

a Requirements are categorized as functions and events (F) and content and structure (C). Each has an identification code (ID) in the first column, matching references in the main text.

b HCP: health care professional.

**Table 8.** Overview of the requirements with justification and priority levels^a^.

ID	Requirement	Justification	Must have	Should have	Could have	Will not have
Functions and events						
F30	The dashboard displays blood pressure and weight.	Displaying blood pressure and weight helps HCPs^b^ to get a broader overview		✓		
F31	The dashboard includes insights into sleep patterns, including total sleep duration and sleep quality.	Insights into sleep-related data to support its impact on glucose regulation		✓		
Content and structure						
C5	The dashboard provides access to medication data, including insulin use.	Insight into medication data including insulin is important to provide actual care, without the need to use HIX^c^ or HIS^d^	✓			
C6	The dashboard integrates results of clinical laboratory tests.	Having access to relevant laboratory results directly within the dashboard enables HCPs to get a complete overview without needing to switch to other systems like HIX or HIS			✓	
C7	The dashboard shows the progress of weight over time.	To give insight into the weight changes			✓	

a Requirements are categorized as functions and events (F) and content and structure (C). Each has an identification code (ID) in the first column, matching references in the main text.

b HCP: health care professional.

c HIX: Healthcare Information Exchange.

d HIS: Huisarts Informatie Systeem (General Practitioner Information System).

**Figure 2.**
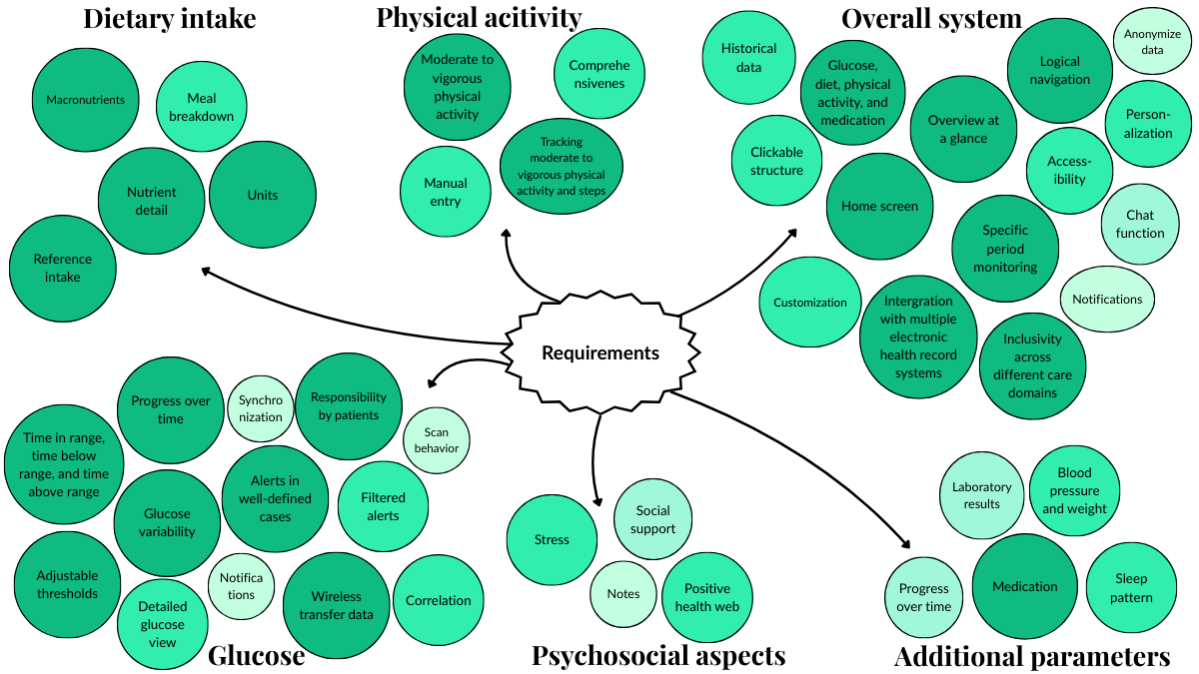
Overview of dashboard requirements.

Figure 2 provides an overview of all identified requirements for the dashboard. The color and size of each element indicate its priority according to the MoSCoW classification [41]; the darker and larger the element, the higher its importance.

### Overall System Requirements

A set of 18 overarching requirements relates to the general functionality, usability, and accessibility of the dashboard, as shown in Table 3. These requirements ensure that the system can be used efficiently by HCPs across different disciplines and settings. In addition, participants emphasized the need to embed the dashboard within existing care pathways. Rather than serving as a stand-alone tool, the dashboard should support blended and transmural care by fostering collaboration across care levels. Participants noted that current diabetes care often lacks integrated digital solutions to monitor and address lifestyle-related factors, underscoring the importance of tools that facilitate communication and shared decision-making.

To support this, the dashboard should be accessible to all HCPs involved in diabetes management, including diabetes nurses, primary care physicians, and specialists (F1). Interoperability was considered essential to enable joint monitoring of data of persons with diabetes, establish personalized goals with the person with diabetes, and ensure seamless transitions between primary and secondary care. To achieve this and ensure integration with existing care pathways, the dashboard should be able to connect to existing software systems (I1) and multiple electronic health record systems (I2), as different care levels typically use different platforms. For sustainable implementation, it is also important that the dashboard can connect to additional health apps commonly used by persons with diabetes (F2). A central component is the home screen (F3), which should provide an accessible and simplified overview of key parameters relevant for the individual HCP (S1). Participants identified glucose values, nutritional intake, physical activity, and medication use as the most important parameters for this overview (F4), which will be explained in more detail later. To support quick interpretation and minimize unnecessary complexity, information should be presented in a concise and straightforward manner (I3): “It shouldn’t be too much, of course, otherwise you won’t get a quick overview” (Participant 1, focus group 2).

From this overview, HCPs should be able to easily navigate to more detailed data if needed. This clickable structure (F5) supports quick access to relevant measurements without overwhelming the user:

*You should be able to click through it quickly. And if you really want to zoom in, like on a particular day, to see what exactly happened, maybe that requires a few more clicks. But even then, you should instantly get digestible averages and values to discuss with patients.*

[Participant 1, focus group 1]

To prevent information overload and enhance relevance, the home screen should be customizable. HCPs should be able to select which parameters are displayed, based on personal goals (eg, exercise more) and/or circumstances (eg, comorbidities) of each person with diabetes (I4). In line with this, the dashboard should allow users to monitor the progress of persons with diabetes over a specific time period (F6). This prevents overload from entering data for persons with diabetes and from reviewing extensive information for HCPs.

*Certain key point for these four months. So not everything necessarily needs to be tracked all the time, some aspects should always be monitored, while others will be the primary focus for the upcoming four months.*

[Participant 5, focus group 2]

Additionally, historical data should be easy to navigate and visually structured, allowing HCPs to switch between daily, weekly, or monthly views depending on the context (S2). Frequently used functionalities should be directly accessible, and overall navigation should remain intuitive to support clinical workflows without adding burden (I5).

Participants also mentioned the importance of communication between persons with diabetes and HCPs. They suggested that a built-in chat function (F7) could serve as a replacement for the email communication currently used. However, one HCP mentioned that without automatic notifications, they would have to continuously proactively check the chat, which is not feasible (F8): “But then as a healthcare provider, you really need to receive an email or something, otherwise you have to check everything manually, which just doesn’t work” (Participant 2, focus group 2).

A potential future feature for the dashboard was mentioned to send persons with diabetes personalized messages based on their physical activity, nutrition, and glucose data using artificial intelligence (AI; F9). Finally, participants would appreciate having an option to anonymize data for training or research purposes available (F10).

### Requirements Regarding Monitoring Dietary Intake

Five requirements concern the monitoring of nutritional data, as shown in Table 4. These include the ability to provide an overview of consumed carbohydrates, amounts of fat, and proteins (F11), representing the core nutritional metrics needed for basic monitoring. There is also a need to present this information per meal moment (breakfast, lunch, dinner, and in-between meals; F12) and as daily totals in grams (C2). The focus groups further indicated that HCPs would appreciate a more comprehensive and detailed breakdown of nutritional data beyond these core metrics. Specifically, they suggested including additional aspects such as fluid consumption and alcohol use, alongside the existing breakdown of macronutrients (C3). This level of detail is considered necessary to support more targeted dietary counseling.

Another key theme that emerged from the focus groups was the lack of confidence among HCPs in providing nutritional advice, particularly when persons with diabetes ask specific or complex questions. As one participant reflected:

*I sometimes find it difficult to give good advice in this area, because I don’t have a background in dietetics. And sometimes people have very specific questions, and I think, oh, yes ... but I don’t really have the exact answer either. ... I’m still trying to figure out what I can do with it [the nutritional data].*

[Participant 1, focus group 1]

and

*As doctors, at least that’s how I experience it, I really feel that I lack sufficient knowledge when it comes to nutrition. I just don’t think I know enough to give proper advice on it.*

[Participant 4, focus group 1]

In response to these concerns, the system should provide easily accessible information about nutritional guidelines (F13), such as the percentage of dietary reference intake for relevant nutrients (see F11). However, one participant also questioned whether it is truly the role of HCPs in secondary or primary care to provide such detailed nutritional information, considering that nutrition is a specialized field. The participant suggested that HCPs might only need to have a certain basic level of knowledge. Such remarks do not directly translate into design requirements for the dashboard but rather highlight important implementation considerations regarding professional roles and training needs.

### Requirements for Physical Activity

Four requirements relate to the presentation and interpretability of physical activity data within the dashboard, as shown in Table 5. HCPs in the focus groups expressed a preference for metrics that are easy to interpret and actionable in clinical practice. They specifically preferred using step counts and minutes of moderate to vigorous physical activity rather than calorie-burn data (F14): “Minutes of exercise, moments, frequency during the week, and minutes” (Participant 3, focus group 1).

It was recommended that both step counts and moderate to vigorous physical activity be displayed across multiple time frames (daily, weekly, monthly, and yearly; F15), enabling HCPs to track progress over time. In addition, HCPs emphasized the importance of being able to view other types of physical activity, such as swimming or cycling (F16). To ensure a complete picture of physical activity, HCPs also recommended including an option for persons with diabetes to manually add activities that may not be captured by standard trackers (I6).

### Requirements for Glucose Values

A total of 14 requirements concern the monitoring of glucose values, as shown in Table 6. To provide personalized advice on glucose levels, it is essential for HCPs to have access to a recent (ie, approximately the past 2‐4 weeks) and clearly presented overview of glucose data, reflecting the most current measurements of persons with diabetes (I7). Therefore, glucose values must be prominently displayed on the home screen of the dashboard, including key indicators such as the percentage of time in range, time below range, and time above range (F17), as well as trends over time (F18).

The clickable detailed view should offer deeper insight into the glycemic patterns of persons with diabetes. It must display daily glucose variability (F19) and clearly illustrate how lifestyle factors such as nutrition, physical activity, and medication intake influence glucose levels over time (S3). As one participant emphasized: “That you basically just hover over the [glucose] curve and then have those [nutrition, activity, medication] data” (Participant 4, focus group 2).

The use of AI to adjust medication based on glucose sensor data was identified as a potential future feature (F20). The focus groups also highlight the importance of wireless transfer of glucose data to the dashboard (I8), synchronization with various glucose monitors (F21), and the option to adjust blood glucose thresholds to allow for individualized targets (F22).

Additional features included displaying how frequently persons with diabetes scan their glucose sensors (F23). This information helps HCPs identify potential issues such as excessive scanning, which could indicate increased stress or anxiety related to glucose monitoring. Notifications with general advice should be sent to the person with diabetes when glucose values are out of range (F24). Regarding forwarding alerts to HCPs, participants agreed that this should only happen in specific, well-defined cases (F25), with persons with diabetes remaining primarily responsible for acting on their glucose data (I9). Alerts should furthermore be filtered according to predefined clinical protocols to avoid unnecessary notifications (F26).

### Requirements for Psychosocial Aspects of Living With T2DM

Four requirements address the role of the social domain in T2DM management, as shown in Table 7. Participants emphasized that both persons with diabetes and HCPs often lack insight into available social support:

*The patient usually doesn’t know where to go.*

[Participant 2, focus group 2]

*And neither do we.*

[Participant 5, focus group 2]

This shared lack of awareness underscores the need for better integration of social support information in clinical practice. To address this, HCPs suggested incorporating regional social domain information into the dashboard (F27). This would enable providers to more easily access and refer to relevant resources.

Beyond informational access, HCPs also emphasized the direct influence of social circumstances on diabetes management. Stress was specifically highlighted as an essential factor (F28), with the social environment, including work and home life, impacting glucose levels and well-being.

*Stress factors, social home situation. Just how things are going.*

[Participant 2, focus group 1]

Similarly, another participant stressed the relevance of mental well-being as a key aspect of the social domain:

*And I do ask about mental well-being as well, because it is one of the first aspects to assess within social factors.*

[Participant 4, focus group 1]

To integrate these dimensions into the dashboard, HCPs suggested using the Positive Health spider web [45] as a framework (C4). This tool offers a structured way to assess various aspects of well-being, making it easier to identify challenges and provide targeted support. Additionally, HCPs expressed a desire for persons with diabetes to have the ability to add personal notes within the dashboard to contextualize their glucose data, particularly in relation to social or emotional challenges (F29).

### Additional Parameter Requirements

HCPs emphasized that T2DM management requires attention to the broader cardiometabolic risk profile. In line with this, HCPs highlighted the need to access up-to-date medication overviews (C5), integration of laboratory results such as hemoglobin A_1c_ levels and blood- and urine cultures (C6), and the visualization of blood pressure and weight (F30), as shown in Table 8: “And furthermore, it would be nice if, across the entire dashboard, you could also include parameters like blood pressure and weight” (Participant 1, focus group 1).

Weight was identified as an important health parameter to monitor, as weight loss is known to reverse the underlying metabolic abnormalities of type 2 diabetes and thereby improve glucose control [46]. To support this process, HCPs noted that weight trends should be displayed over time to enable monitoring of long-term progress (C7). Many people with diabetes also have hypertension. Elevated blood pressure combined with diabetes increases the risk of cardiovascular and renal complications, making routine monitoring highly relevant for clinical decision-making [47].

Sleep was also considered potentially informative, as poor sleep can negatively affect glucose regulation and overall well-being. Tracking indicators such as total sleep time and sleep disturbances (F31) may therefore offer additional insight, although participants differed in how essential they perceived this parameter to be.

## Discussion

### Principal Findings

This study aimed to identify requirements for a personalized blended dashboard for T2DM care, based on the perspectives of HCPs. The aim was to facilitate informed decision-making with persons with diabetes, enabling more individualized care with personalized goals in a blended care setting. The findings reveal strong support for integrating key lifestyle parameters such as physical activity, dietary intake, glucose values, and psychosocial factors, as well as overall system requirements and additional health-related parameters (eg, medication use and sleep). They underscore the need for an integrated approach to lifestyle monitoring in diabetes care, recognizing the interaction between behaviors such as diet, physical activity, glucose levels, and psychosocial factors. This reflects a growing recognition in the literature that behavioral and emotional contexts play a critical role in diabetes self-management [48-51]. Integrating lifestyle and behavioral data into care underscores the need for shared decision-making tools that facilitate collaboration between patients and HCPs and support personalized diabetes treatment.

### Findings in Context

The need for dashboards supporting personalized and blended care is also seen in the literature. Existing shared decision-making tools and/or decision support tools for T2DM address a variety of aspects. Some focus on medication-related decisions, including guidance on drug interactions, dosage adjustments, or therapy changes [52-56]. Others incorporate lifestyle-related data [57-59], including structured forms for diet, physical activity, glucose data, and self-management. However, these tools rarely facilitate the collaborative setting of personalized lifestyle goals with persons with diabetes. For example, the integrated personalized diabetes management system [58] involves persons with diabetes in therapy adjustments based on self-measured blood glucose, but does not integrate diet or physical activity into home monitoring and provides limited support for lifestyle goal setting. Similarly, the Smart Care service offers video consultations, remote monitoring, automated feedback, and educational resources [59] without actively guiding personalized lifestyle decisions. Compared to these existing tools, the requirements identified in our focus groups emphasize offering concrete and personalized lifestyle-related support and the integration of personal values into decision-making. Two exceptions can be highlighted. MyDiabetesPlan is an online decision aid that supports shared decision-making by assessing the clinical and psychosocial profiles of persons with diabetes, eliciting personal goals, and providing a tailored action plan with concrete strategies to achieve them [60]. However, it does not incorporate home-monitored lifestyle data, limiting its ability to provide truly data-driven lifestyle feedback. Another exception is found in pregnancy care, where a dashboard has been developed for women with T2DM by combining clinical data, lifestyle monitoring, and patient-centered decision support [61]. However, as far as we know, no dashboard currently exists for the broader T2DM population that integrates home-monitored lifestyle behaviors with clinical data. Such a solution would ideally provide patient-centered decision support in a single, personalized tool suitable for use in a blended care pathway, preferably within a single uniform platform that can be used across both primary and secondary care.

International guidelines for the treatment of T2DM emphasize the importance of lifestyle advice, highlighting the crucial role of a healthy lifestyle [19,62,63]. However, translating these recommendations into daily practice remains a challenge, as reflected by our focus groups, where HCPs expressed uncertainty about referrals in the social domain and lacked knowledge in providing personalized nutrition consultations. This highlights the need for clear and accessible dashboard interfaces that integrate clinical guidelines with health data of persons with diabetes. In addition to that, it shows a need for ongoing professional development and training. In the Netherlands, initiatives such as the “Dutch Coalition for Lifestyle in Care” and the “Association for Physicians and Lifestyle” are trying to address this need by promoting structured education and embedding lifestyle medicine more firmly in routine care [64]. At the same time, broader national infrastructure adjustments are required. The integration of lifestyle medicine into existing pathways must be supported by new collaborative structures that enable multiple HCPs to contribute. Such collaboration requires patients to actively consent to sharing their data across providers, and this process is still rarely implemented in current practice [65].

HCPs emphasized that the dashboard must align with their workflow and clinical responsibilities. This reflects a broader challenge in implementing digital tools in routine care, as such tools are most effective when they support rather than disrupt clinical processes [66,67]. Tailoring the information of persons with diabetes to individual needs and integrating features such as alert or notification systems can enhance efficiency and safety of persons with diabetes. At the same time, HCPs stressed that automated alerts should not shift accountability away from persons with diabetes, highlighting the need to balance technological support with responsibility of persons with diabetes.

This study focused on the perspective of HCPs to identify requirements for a dashboard supporting collaborative goal setting and shared decision-making. While this provided important insights into clinical workflows, it must be acknowledged that shared decision-making is inherently bidirectional and requires the active involvement of persons with diabetes. Given that persons with diabetes are expected to monitor their lifestyle and use supportive tools such as continuous glucose monitors or activity trackers, it is essential to incorporate their perspectives to promote self-management and strengthen shared decision-making [68]. Previous work by colleagues from our research group, such as the study by den Braber et al [69], has highlighted the patients’ perspective. This allows us to focus discussions in this study on HCPs, while bringing the different perspectives together again in future work. At the same time, practical considerations such as accessibility, availability, and reimbursement of these tools must be addressed to ensure that sufficient and reliable data can be collected for clinical use [70,71].

### Implications for Practice

The findings indicate that strengthening lifestyle care requires integration of lifestyle medicine into routine practice. Moreover, care pathways should be redesigned to center around a lifestyle counter that enables interdisciplinary collaboration and sustainable use of digital tools. Rather than proposing an entirely new pathway, a complementary model could be envisioned, in which a lifestyle counter team supports existing care structures by coordinating persons with diabetes guidance, monitoring, and follow-up. This approach may be particularly valuable in secondary care settings, where persons with diabetes often have completed a lifestyle intervention, such as a Combined Lifestyle Intervention [72]. In such a model, the dashboard would integrate seamlessly into the workflow by guiding persons with diabetes to appropriate professionals, managing alerts, and coordinating follow-up across medical, dietary, psychosocial, and community-based services. To support this approach, the Dutch Coalition for Lifestyle in Care offers information cards that provide practical guidance and resources for implementing Lifestyle Front Office [73,74]. Embedding the dashboard within such a Lifestyle Front Office ensures that lifestyle monitoring becomes a structurally integrated element of diabetes care while reducing workload for individual providers [75].

This study highlights the need for a dashboard that provides insight into personalized blended care for type 2 diabetes, going beyond clinical data alone. As is good practice in any brainstorm situation, and given the fact that the aim of this paper was merely to explore any potential requirements for the dashboard, no requirements were considered “inappropriate” or “unfeasible” in this study. However, we recognize that not all requirements are equally feasible to implement. For example, HCPs suggested a customizable home screen, which is relatively straightforward to implement, whereas more advanced features, such as AI-driven medication adjustment suggestions based on glucose data, may face technical, cost, or ethical constraints. Although medication management did not emerge prominently in the focus groups, integrating medication-related functionalities into future dashboard versions will be important to create a more comprehensive tool for T2DM care. Assessing feasibility and acceptability is therefore an important consideration for future work.

### Strengths and Limitations

A key strength of this study is the inclusion of health care providers with diverse professional backgrounds, which enhanced the comprehensiveness of the findings. Despite this, several limitations should be acknowledged. Given the exploratory nature of this study, where the goal is to general conceptual insights rather than achieve statistical power, using a relatively small number of participants was fitting and justifiable [40]. However, this does imply that the generalizability of the findings is limited. In addition, female participants were overrepresented, which may introduce bias. However, sex-related effects could not be assessed separately from professional role. Moreover, focus group dynamics may have influenced the prioritization of requirements even though researchers prioritized monitoring and guiding the discussion process throughout the focus group to minimize risk. Despite its potential limitations, the study provides valuable insights into the needs and expectations of HCPs regarding a dashboard tailored to the treatment of people with T2DM. By translating these insights into concrete functional and contextual requirements, this study offers a foundation for the development of a dashboard that can strengthen lifestyle support in diabetes care and facilitate more integrated, multidisciplinary collaboration.

### Conclusions

This study identified key requirements for a remote patient monitoring dashboard for personalized blended diabetes care, emphasizing the integration of data-driven lifestyle parameters alongside clinical data to support shared decision-making and individualized care. The findings highlight strong support for integrating physical activity, dietary intake, glucose levels, psychosocial factors, and additional parameters (eg, weight and blood pressure) to enable an integrated approach to T2DM management. At the same time, the study underscores both the promise and the challenges of translating lifestyle monitoring into routine diabetes care. This includes the need for clear and accessible visualization of lifestyle data, seamless integration into clinical workflows, and professional training in domains such as nutrition and support and psychosocial referral options. A meaningful dashboard must therefore be clinically relevant and user-friendly but also embedded within broader structural changes in health care practice and professional education. This includes multidisciplinary access and alignment with professional guidelines. Addressing both functional and contextual requirements, alongside the development of fitting care pathways, is essential to ensure that a dashboard truly supports HCPs in delivering integrated and personalized lifestyle care.

## Abbreviations

AI: artificial intelligence
HCP: health care professional
MoSCoW: must/should/could/will not have
T2DM: type 2 diabetes mellitus
